# Clinically Interpretable Deep Learning for Differentiating Vitiligo and Postinflammatory Hypopigmentation: Diagnostic Accuracy Study

**DOI:** 10.2196/81942

**Published:** 2026-07-24

**Authors:** Amal Adel Alzu'bi, Shadi Al Khateeb, Bassam M Elzaghmouri, Diala M Alshiyab, Sanaa Abu Alasal, Leming Zhou

**Affiliations:** 1 Department of Computer Information Systems, Faculty of Computer & Information Technology, Jordan University of Science and Technology, Irbid, 22110, Jordan, 962 790051705; 2 Department of Computer Networks, Jerash University, Jerash, Jordan; 3 Department of Data Science and Artificial Intelligence, Al-Ahliyya Amman University, Amman, Jordan; 4 Department of Dermatology, Jordan University of Science and Technology, Irbid, Jordan; 5 Department of Computer Information Systems, Jordan University of Science and Technology, Irbid, Jordan; 6 Department of Health Information Management, University of Pittsburgh, Pittsburgh, PA, United States

**Keywords:** artificial intelligence, skin disease, vitiligo, postinflammatory hypopigmentation, deep learning, explainable AI

## Abstract

**Background:**

Distinguishing vitiligo from postinflammatory hypopigmentation (PIH) is clinically challenging because both conditions may present with similar depigmented lesions. Although deep learning has shown strong potential for dermatologic image classification, limited interpretability remains a barrier to clinical adoption.

**Objective:**

This study aimed to develop an interpretable deep learning framework for accurate differentiation between vitiligo and PIH using a lightweight convolutional neural network and an ensemble of explainability methods.

**Methods:**

A total of 332 clinical images (176 vitiligo and 156 PIH) were collected from King Abdullah University Hospital and publicly available online sources. Images were preprocessed and evaluated using patient-wise 5-fold cross-validation to eliminate patient-level data leakage. A pretrained MobileNetV2 model was fine-tuned by unfreezing the final 30 layers. To enhance interpretability, gradient-weighted class activation mapping (Grad-CAM), integrated gradients, and smooth gradients (SmoothGrad) were combined into an equal-weight ensemble explanation framework. Performance was assessed using accuracy, precision, recall, *F*_1_-score, and the area under the receiver operating characteristic curve (AUC).

**Results:**

The proposed model achieved an overall accuracy of 94.88%, macroaveraged precision of 94.88%, recall of 94.84%, *F*_1_-score of 94.86%, and an AUC of 0.9885 across the 5 validation folds. The ensemble framework produced clinically meaningful explanations in 98.48% of a representative 66-image validation subset used for interpretability analysis.

**Conclusions:**

The proposed framework combines high diagnostic performance with robust interpretability for distinguishing vitiligo from PIH. By integrating multiple complementary explanation methods, the approach enhances clinical transparency and may support dermatologists in the differential diagnosis of pigmentary disorders.

## Introduction

### Background

Skin disorders are a leading global health issue, affecting approximately 1.9 billion people and representing the fourth leading cause of nonfatal disease burden [1]. They encompass a wide range of conditions that often exhibit similar visual patterns, making timely and accurate diagnosis difficult [2,3]. Two such conditions, vitiligo and postinflammatory hypopigmentation (PIH), frequently present with overlapping features, namely localized depigmented patches [4]. However, their etiologies differ significantly. PIH develops as a secondary reaction to skin inflammation, damage, or healing [4], whereas vitiligo is an autoimmune disorder characterized by the loss of melanocytes [5]. Accurately distinguishing between these disorders remains a clinical challenge, particularly in the early stages. Misdiagnosis can lead to inappropriate treatment and unnecessary psychological stress for patients.

Visual examination and clinical history play a major role in the traditional clinical diagnosis of these hypopigmented disorders [4]. However, this approach is inherently subjective and may result in misguided diagnoses, particularly in resource-limited settings [4]. Recent dermatological research has highlighted the diagnostic complexity of pigmentary disorders, particularly when different conditions present with similar patterns of hypopigmentation or depigmentation. Variability in lesion morphology, anatomical location, skin phototype, and disease stage can substantially affect visual appearance and complicate clinical differentiation [6]. In particular, distinguishing vitiligo from PIH may be challenging because both conditions can exhibit well-demarcated pale patches with overlapping textural and pigmentary characteristics. These challenges underscore the need for objective and interpretable AI tools to support clinical decision-making and improve diagnostic consistency [7]. AI-based diagnostic tools offer promising support for addressing these challenges, particularly as digital imaging and necessary computational resources for digital image processing become more widely accessible [8].

Deep learning has emerged as a powerful tool in medical image analysis, particularly in dermatology, where it enables automated classification, segmentation, and anomaly detection with performance approaching that of human specialists [9,10]. In particular, convolutional neural networks (CNNs) have demonstrated remarkable effectiveness in skin lesion analysis, achieving dermatologist-level accuracy in various diagnostic tasks [9,11]. Furthermore, advanced deep learning systems have shown strong capability in the differential diagnosis of skin diseases, highlighting their potential in supporting clinical decision-making [12]. Despite these advancements, the opaque nature of deep learning models, often referred to as “black-box” behavior, remains a significant barrier to their widespread clinical adoption, especially in sensitive conditions such as vitiligo diagnosis [13].

The opaque decision-making process of CNNs raises concerns, particularly in domains such as dermatology, where interpretability is essential rather than optional [14]. To address this limitation, recent studies have integrated explainable AI (XAI) methods with deep learning models [14]. XAI techniques aim to improve model trustworthiness and clinical applicability by visualizing and interpreting the internal operations of neural networks [15]. Gradient-weighted class activation mapping (Grad-CAM) [16], integrated gradients [17], and smooth gradients (SmoothGrad) [18] are among the most widely used methods in this field, each providing a different perspective on model decision-making by highlighting relevant regions of input images [16].

### Objective

Despite advances in XAI, individual saliency-based methods often exhibit noise, instability, and sensitivity to model architecture and parameter settings [16]. To address these limitations and generate more consistent and clinically meaningful explanation maps, we propose an ensemble interpretability framework that combines Grad-CAM, integrated gradients, and SmoothGrad.

In this study, we present an interpretable deep learning framework for differentiating vitiligo and PIH using a fine-tuned MobileNetV2 architecture [19]. The model was trained on dermatological images collected from both hospital and publicly available sources and evaluated using a patient-wise 5-fold cross-validation strategy. By integrating 3 complementary explainability methods, the proposed framework aims to improve the robustness of visual explanations and enhance the model’s trustworthiness for potential use in clinical decision support systems.

### Literature Review

Deep learning has shown considerable promise in automating dermatological diagnoses, particularly for conditions such as vitiligo [4,13,20-22]. However, the clinical reliability of these models depends not only on their classification accuracy but also on their interpretability and generalizability across diverse populations and dermatological conditions.

Several studies have addressed the classification of vitiligo using deep learning, tackling challenges such as data heterogeneity, image acquisition inconsistencies, and computational efficiency. For instance, Kantoria et al [23] used a dataset of 696 images and applied transfer learning with models such as Inception-V3 [24], Visual Geometry Group (VGG)-16, VGG-19 [25], and SqueezeNet [26], followed by classifiers such as k-nearest neighbors, support vector machine, and logistic regression. Although Inception-V3 with logistic regression achieved the highest accuracy (98.0%), the method was computationally intensive, limiting its applicability in real-time settings. Similarly, Guo et al [21] adopted a You Only Look Once (YOLO) v3–based hybrid model using 2720 images for lesion classification, yet reported performance issues in detecting lesions near anatomical boundaries and challenges stemming from label noise.

To enhance image clarity, Luo et al [27] proposed a Cycle-Consistent Adversarial Network (CycleGAN)–based model incorporating super-resolution techniques, achieving a classification accuracy improvement from 76.37% to 85.69%. Despite this gain, the method’s high computational demand posed a practical limitation for resource-limited settings. Zhang et al [28] investigated multiple CNN architectures, including VGG-13, residual network (ResNet)-18, and Densely Connected Convolutional Networks (DenseNet)-121 [29], across in-house and public datasets. Their models outperformed dermatologists in certain tasks; however, the datasets were demographically limited to Asian women and children, thus constraining the models’ generalizability.

Although deep learning–based classification has been extensively explored for dermatological conditions, including hypopigmented dermatoses, no previous study has explicitly addressed the PIH using image-based techniques with visual interpretability. Several works have proposed visual explanation tools such as Grad-CAM, Grad-CAM++ [30], and Score-CAM [31] for skin cancer diagnosis. While these contributions have advanced the field of XAI in dermatology, none have applied these frameworks specifically to distinguish between vitiligo and PIH using clinical images.

Complementing the classification-focused studies, other research has emphasized XAI as a means to foster trust in medical diagnostics. For example, Badhon et al [32] used Grad-CAM with transfer learning on a balanced dataset, achieving 97.07% accuracy while enhancing transparency through saliency maps. Zhong et al [22] incorporated class activation mapping (CAM) with Swin Transformer models [33], visually highlighting lesion areas, though not always aligning with clinical boundaries. Shorfuzzaman [34] proposed a Shapley additive explanations (SHAP)-enhanced stacked CNN ensemble for melanoma detection, while Wang et al [10] introduced a multimodal interpretability-based CNN incorporating Grad-CAM for image features and SHAP for patient metadata to improve clinical trust.

Despite these advancements, challenges remain concerning real-time feasibility, dataset diversity, and the robustness of explanation techniques. These gaps highlight the need for lightweight, interpretable, and clinically relevant deep learning workflows. Our study addresses this need by introducing a MobileNetV2-based model designed for efficient and real-time inference, coupled with an ensemble visual explanation framework that integrates Grad-CAM, integrated gradients, and SmoothGrad. To the best of our knowledge, this is the first attempt to differentiate vitiligo and PIH using explainable deep learning on clinical images, bridging a critical gap in dermatological AI.

## Methods

In this study, we developed a robust and interpretable deep learning framework for differentiating skin images of vitiligo and PIH, 2 conditions that are clinically and visually difficult to distinguish. The proposed workflow comprised three major stages: (1) preprocessing input images to meet model requirements, including resizing, normalization, and offline augmentation; (2) training and fine-tuning a MobileNetV2-based classifier using patient-wise 5-fold cross-validation; and (3) applying 3 explainability techniques—Grad-CAM, integrated gradients, and SmoothGrad—to provide interpretable visual explanations of the model’s predictions.

### Ethical Considerations

Ethical approval was granted by the King Abdullah University Hospital (KAUH) research ethics committees (approval number 50/161/2023, date: June 8, 2023). The requirement for informed consent was waived due to the retrospective nature of the study and the use of anonymized data.

### Dataset Description and Preprocessing

The image dataset comprised 2 classes: vitiligo and PIH. A total of 332 clinical images were included, consisting of 176 vitiligo images obtained from KAUH in Irbid, Jordan, and 156 PIH images collected from KAUH and publicly accessible online sources. All images were labeled and verified by dermatologists.

**Figure 1.**
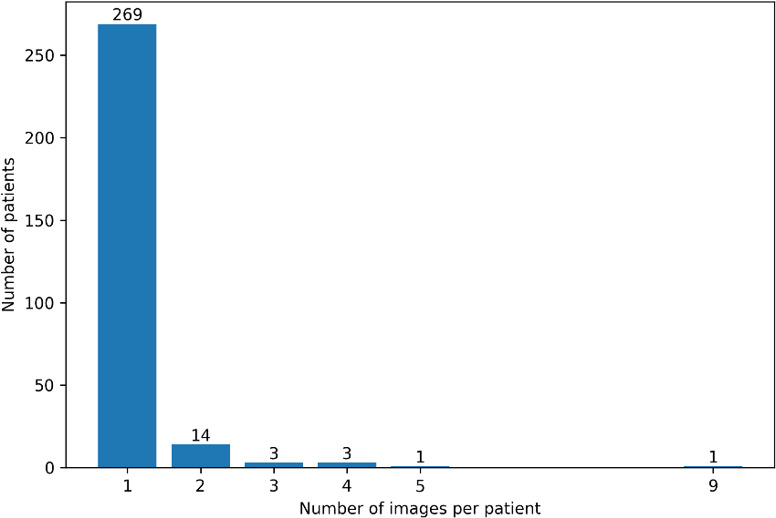
Distribution of the number of images per patient in the final dataset.

The final dataset comprised 332 images obtained from 291 unique patients. Most patients contributed a single image (median=1 image per patient), while the mean number of images per patient was 1.14 (SD 0.66). The maximum number of images contributed by a single patient was 9. Figure 1 summarizes the full distribution of images per patient. These statistics indicate that the majority of patients were represented by only 1 image, although a small number of patients contributed multiple images from different anatomical sites.

To minimize potential source-related bias arising from mixed image origins, all images were processed using a unified preprocessing pipeline, including resizing to 224×224 pixels, pixel-value normalization, and identical augmentation operations. Although a metadata file was used to organize image labels and patient identifiers for patient-wise cross-validation, no metadata or acquisition-related variables were provided as input features to the model. The classifier was trained exclusively on image pixel data.

The dataset was organized at the patient level, with each image linked to a unique patient identifier. Multiple images from the same patient were retained when they captured anatomically distinct lesions or body regions. During model evaluation, a patient-wise 5-fold cross-validation strategy was employed, ensuring that all images from a given patient were assigned exclusively to either the training or validation subset within each fold. This prevented patient-level data leakage and provided a more rigorous estimate of generalization to previously unseen patients.

Offline data augmentation was applied exclusively to the training subset within each fold and included random rotations, horizontal and vertical flips, brightness adjustments, and zooming operations. For each original training image, 5 augmented variants were generated, resulting in a 6-fold increase in the effective training dataset size, including the original image. The corresponding validation images were left unchanged to ensure unbiased evaluation within each fold. Because of the limited dataset size, no separate holdout or external test set was used; all performance metrics were computed exclusively from the validation folds generated during the patient-wise 5-fold cross-validation procedure.

### Model Architecture and Training Strategy

Given the need for a lightweight yet high-performing model, MobileNetV2 was selected as the backbone architecture. MobileNetV2 is a CNN optimized for mobile and embedded vision applications. The base model, pretrained on ImageNet [35], was adapted using transfer learning [36]. Initially, all backbone layers were frozen to leverage general visual features such as edges and textures. A subsequent fine-tuning phase was performed by unfreezing the final 30 layers of the network, specifically from block_13_expand to out_relu, enabling adaptation to dermatological features specific to vitiligo and PIH.

A custom classification head was appended to the convolutional base. This head consisted of a GlobalAveragePooling2D layer, followed by a fully connected dense layer with 128 units and ReLU activation, a dropout layer with a rate of 0.3 to reduce overfitting, and a final sigmoid-activated output layer for binary classification.

The model was trained using the Adam optimizer and binary cross-entropy loss. During the initial frozen training phase, the learning rate was set to 1×10⁻⁴. During fine-tuning, the learning rate was reduced to 1×10⁻⁵ to preserve pretrained features while enabling gradual adaptation of the unfrozen layers. Training was performed for up to 25 epochs with a batch size of 32 and early stopping based on validation loss (patience=5). Hyperparameters, including learning rate, batch size, and the number of trainable layers, were selected empirically.

Model performance was evaluated using patient-wise 5-fold cross-validation. Aggregated predictions across all validation folds were used to compute the final accuracy, precision, recall, *F*_1_-score, and area under the receiver operating characteristic curve (AUC). This approach provided a robust estimate of the model’s diagnostic performance on previously unseen patients. Hyperparameters used in the model are presented in Table 1.

**Table 1.** Hyperparameters used in the MobileNet model and their values^a^.

Hyperparameter	Value	Description
Model architecture	MobileNetV2 (pretrained on ImageNet)	Lightweight convolutional neural network used as the backbone model
Input image size	224×224×3	Standard input dimensions for MobileNetV2
Epochs	25	Maximum number of training epochs
Batch size	32	Number of images processed per batch
Optimizer	Adam	Optimization algorithm
Learning rate	1×10⁻⁴ (initial), 1×10⁻⁵ (fine-tuning)	Learning rates used during frozen training and fine-tuning
Loss function	Binary cross entropy	Loss function for binary classification
Early stopping patience	5	Training stopped if validation loss did not improve for five consecutive epochs
Preprocessing	MobileNetV2 preprocess_input	Scales pixel values to the range [–1, 1]
Data augmentation	Rotation, zoom, horizontal and vertical flips, brightness adjustment	Applied offline to training images only
Fine-tuning	Last 30 layers unfrozen	Layers from block_13_expand to out_relu were retrained
Dense layer units	128	Number of neurons in the fully connected layer
Dropout rate	0.3	Used to reduce overfitting
Evaluation strategy	Patient-wise 5-fold cross-validation	Prevents patient-level data leakage and provides robust performance estimates

a Name, value, and corresponding description of those hyperparameters are listed in the table.

### Ensemble Explanation Framework

To enhance model interpretability, which is essential for the clinical deployment of AI systems, 3 complementary explanation methods were integrated: Grad-CAM [16], integrated gradients [17], and SmoothGrad [18]. Each method captures a distinct perspective of feature importance within the input image.

1. Grad-CAM generates coarse localization maps by computing the gradients of the predicted class score with respect to the activations of the final convolutional layer, thereby highlighting class-discriminative regions.
2. Integrated gradients attribute the model prediction by integrating gradients along a straight-line path from a baseline image to the input image, producing pixel-level attribution scores.
3. SmoothGrad improves the visual quality of gradient-based saliency maps by averaging gradients computed from multiple noisy perturbations of the input image.

To address the limitations of relying on a single explanation technique, an ensemble explanation map was constructed by combining the outputs of the 3 methods. For each input image, the saliency maps produced by Grad-CAM, integrated gradients, and SmoothGrad were resized to a common spatial resolution using bilinear interpolation and normalized to the range [0, 1]. For integrated gradients and SmoothGrad, the absolute magnitude of the attribution values was used prior to normalization to avoid cancelation effects arising from signed gradients and to ensure consistent comparison with the nonnegative Grad-CAM activation maps.

The final ensemble map was obtained by computing the arithmetic mean of the 3 normalized saliency maps, with equal weights assigned to each method:

M_Ensemble(x) = (1/3) × M̂_GradCAM(x) + (1/3) × M̂_IG(x) + (1/3) × M̂_SmoothGrad(x) (1)

where x denotes the input image and M̂_GradCAM(x), M̂_IG(x), and M̂_SmoothGrad(x) represent the normalized saliency maps generated by Grad-CAM, integrated gradients, and SmoothGrad, respectively. The ensemble explanation map M_Ensemble(x) is obtained by computing the arithmetic mean of the 3 normalized maps.

This equal-weight aggregation was adopted to preserve the complementary strengths of the 3 explanation methods while reducing method-specific noise and instability. No adaptive weighting or gating mechanism was applied; therefore, all 3 methods contributed equally to the final ensemble explanation.

### Workflow Implementation

The workflow began with preprocessing the input images to satisfy the model’s input requirements. After training and fine-tuning the MobileNetV2 classifier, Grad-CAM, integrated gradients, and SmoothGrad were applied independently to generate saliency maps for each image.

The 3 maps were subsequently normalized and averaged according to equation (1) to generate the ensemble explanation. The final output provided both the predicted class and a composite visual explanation highlighting the regions that most strongly influenced the model’s decision, thereby improving interpretability and clinical transparency.

## Results

### Performance Improvement Through Fine-Tuning

**Figure 2.**
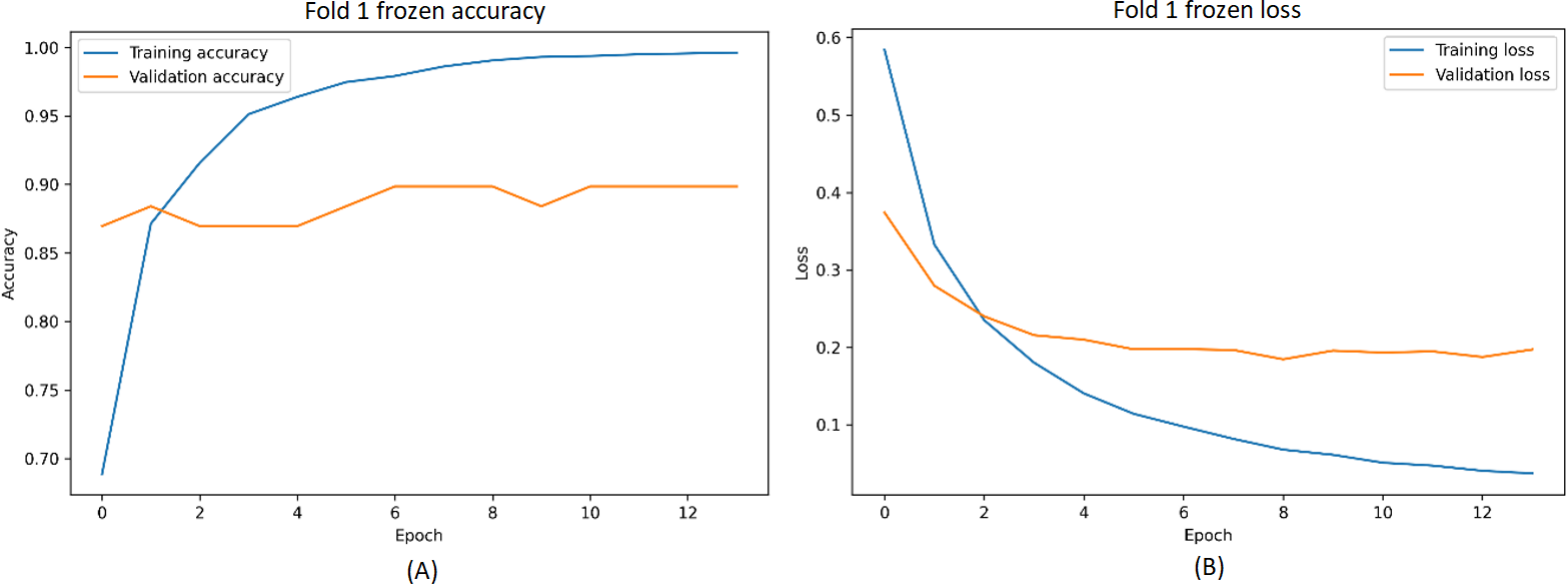
Representative training and validation accuracy and loss curves for fold 1 during the frozen training phase, in which all MobileNetV2 backbone layers were kept nontrainable. Panel (A) shows training and validation accuracy, and panel (B) shows training and validation loss.

Initially, the MobileNetV2 model, with all convolutional layers frozen, served as a strong baseline. Figure 2 presents representative training and validation curves for fold 1 during the frozen training phase, including both accuracy and loss. The corresponding training and validation curves for all 5 folds are provided in Multimedia Appendix 1. During this phase, the model demonstrated good discriminative ability; however, the extracted features remained largely aligned with generic ImageNet representations rather than dermatology-specific characteristics. Consequently, the model showed limitations in distinguishing subtle lesion patterns in visually ambiguous cases.

**Figure 3.**
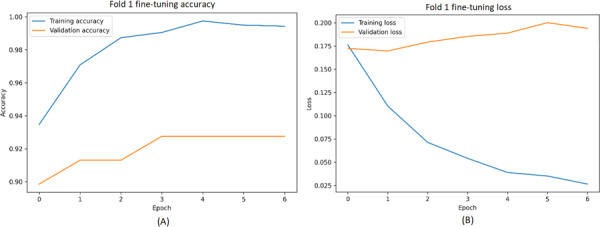
Representative training and validation accuracy and loss curves for fold 1 during the fine-tuning phase, after unfreezing the final 30 layers of the MobileNetV2 backbone. Panel (A) shows training and validation accuracy, and panel (B) shows training and validation loss.

To improve domain adaptation, fine-tuning was performed by unfreezing the final 30 layers of the MobileNetV2 backbone, as described in the *Methods* section, specifically from the block_13_expand layer to out_relu. Figure 3 illustrates representative training and validation curves for fold 1 during the fine-tuning phase, while the corresponding curves for all 5 folds are included in Multimedia Appendix 1.

A temporary increase in validation loss was observed at the beginning of the fine-tuning phase. This behavior is expected because the optimizer state was reinitialized and previously frozen layers were unfrozen, substantially increasing the number of trainable parameters and altering the optimization landscape. As a result, the model required several epochs to readjust the weights before achieving stable convergence.

Following fine-tuning, the model demonstrated improved qualitative and quantitative performance. In particular, the fine-tuned model showed enhanced discrimination between depigmented vitiligo lesions and hypopigmented PIH regions, even in clinically challenging cases. The model appeared to rely on diagnostically relevant features such as lesion border sharpness, pigment uniformity, and contrast with surrounding skin.

Table 2 summarizes the key improvements observed after fine-tuning compared to the frozen baseline.

**Table 2.** Comparative performance characteristics of the MobileNetV2 model before and after fine-tuning.

Metric	Frozen MobileNetV2	Fine-Tuned MobileNetV2
Feature representation	Generic (ImageNet-based)	Domain-specific (vitiligo vs PIH^a^)
Clinical differentiation	Moderate	High
Validation loss	Higher	Reduced
Generalization performance	Good	Improved
Overfitting risk	Low	Low (stable convergence)

a PIH: postinflammatory hypopigmentation.

### Model Evaluation and Performance Analysis

The final model was evaluated using patient-wise 5-fold cross-validation, ensuring that all images from a given patient were assigned exclusively to either the training or validation subset within each fold. This strategy provided a rigorous assessment of model generalization to previously unseen patients.

The number of validation images per fold ranged from 61 to 72, with no patient overlap between training and validation sets. Class balance was preserved across all folds, with each validation fold containing approximately 29‐41 vitiligo images and 30‐32 PIH images.

Across all 5 folds, the model generated 1 prediction for each of the 332 images. Aggregating these predictions yielded an overall accuracy of 94.88% and an AUC of 0.9885, indicating excellent discriminative performance. Table 3 summarizes the class-wise classification metrics. Fold-specific best-validation epochs and stopping epochs for both the frozen training and fine-tuning phases are provided in Table S1 in Multimedia Appendix 1.

These results demonstrate strong and well-balanced diagnostic performance across both classes. The comparable precision and recall values indicate that the model does not exhibit a substantial bias toward either vitiligo or PIH.

**Table 3.** Classification performance of the fine-tuned MobileNetV2 model using patient-wise 5-fold cross-validation^a,b^.

Class	Precision	Recall	*F*_1_-score
PIH^c^	0.9484	0.9423	0.9453
Vitiligo	0.9492	0.9545	0.9518

a Overall accuracy: 0.9488.

b Area under the receiver operating characteristic curve: 0.9885.

c PIH: postinflammatory hypopigmentation.

### Classification Metrics and Diagnostic Performance

**Figure 4.**
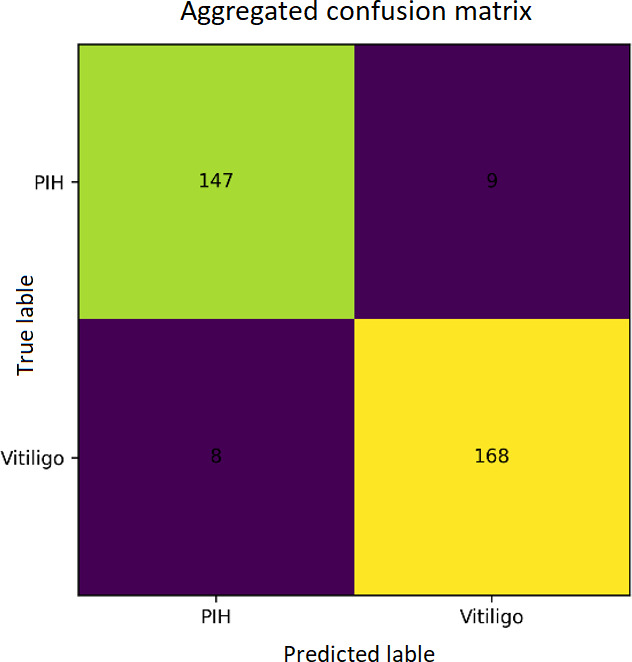
Aggregated confusion matrix of the fine-tuned MobileNetV2 model for differentiating vitiligo from postinflammatory hypopigmentation (PIH). The matrix includes predictions from all 5 validation folds, with each image appearing exactly once.

The aggregated confusion matrix (Figure 4) summarizes the predictions across all 5 validation folds, with each image contributing exactly once to the final evaluation.

The model correctly classified 147 of 156 PIH images and 168 of 176 vitiligo images, resulting in only 17 misclassifications across the entire dataset. Specifically, 9 PIH images were incorrectly classified as vitiligo, whereas 8 vitiligo images were incorrectly classified as PIH.

The relatively small and nearly symmetric number of false-positive and false-negative predictions further confirms the balanced classification behavior of the model and supports its potential use for differentiating these visually similar pigmentary disorders.

**Figure 5.**
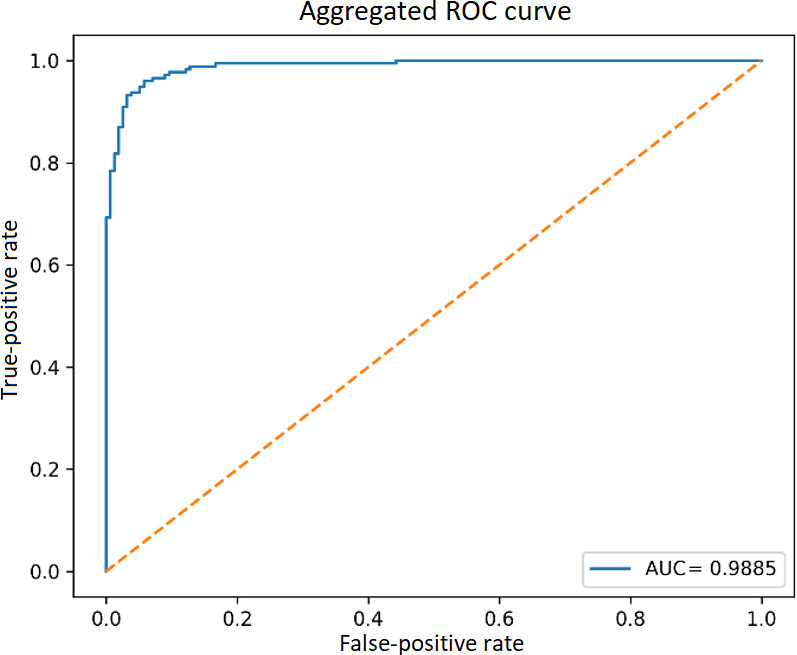
Aggregated receiver operating characteristic (ROC) curve of the fine-tuned MobileNetV2 model across all 5 validation folds. The dashed diagonal line represents random classification performance (area under the receiver operating characteristic curve [AUC]=0.50).

The receiver operating characteristic (ROC) curve aggregated across all 5 validation folds (Figure 5) yielded an AUC of 0.9885, demonstrating excellent separability between vitiligo and PIH.

### Model Interpretability Through Ensemble Explanation Methods

#### Overview

**Figure 6.**
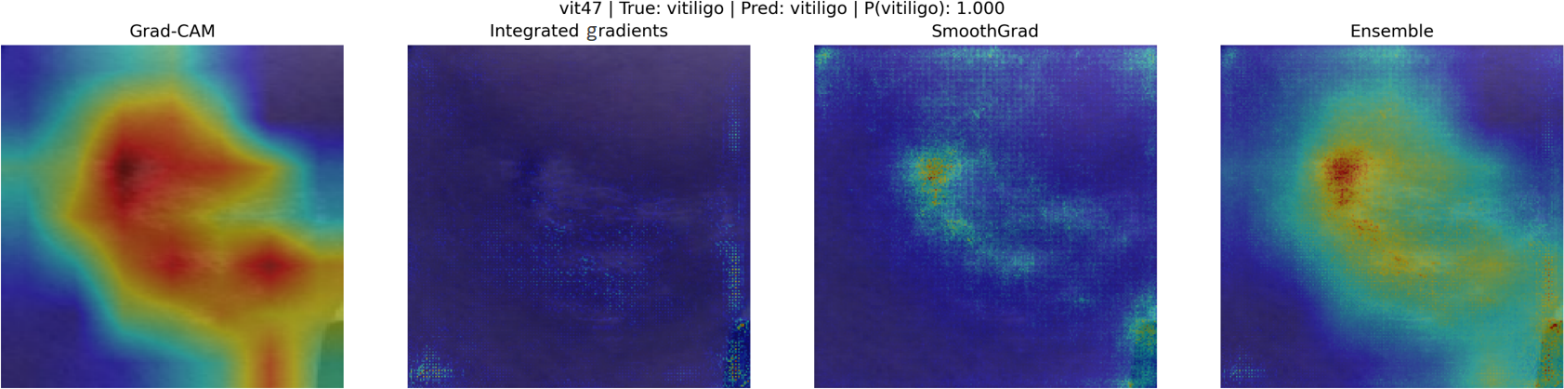
Representative example of scenario 1, where gradient-weighted class activation mapping (Grad-CAM), integrated gradients, smooth gradients (SmoothGrad), and the ensemble map consistently highlighted the same lesion region, demonstrating strong agreement across all explanation methods.

**Figure 7.**
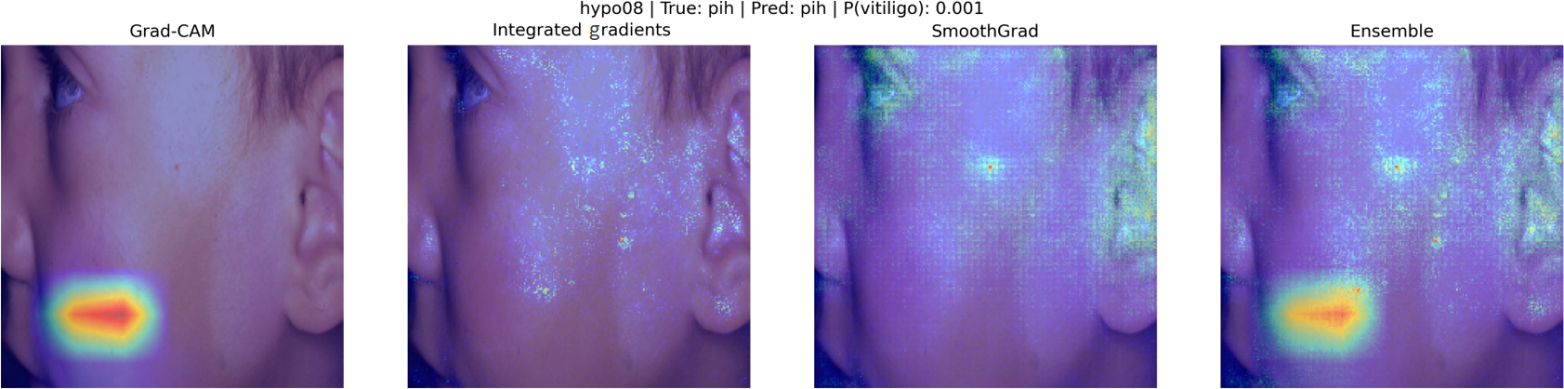
Representative example of scenario 2, where 1 explanation method produced a suboptimal attribution map, whereas the ensemble map remained robust and accurately localized the lesion. Grad-CAM: gradient-weighted class activation mapping; SmoothGrad: smooth gradients.

**Figure 8.**
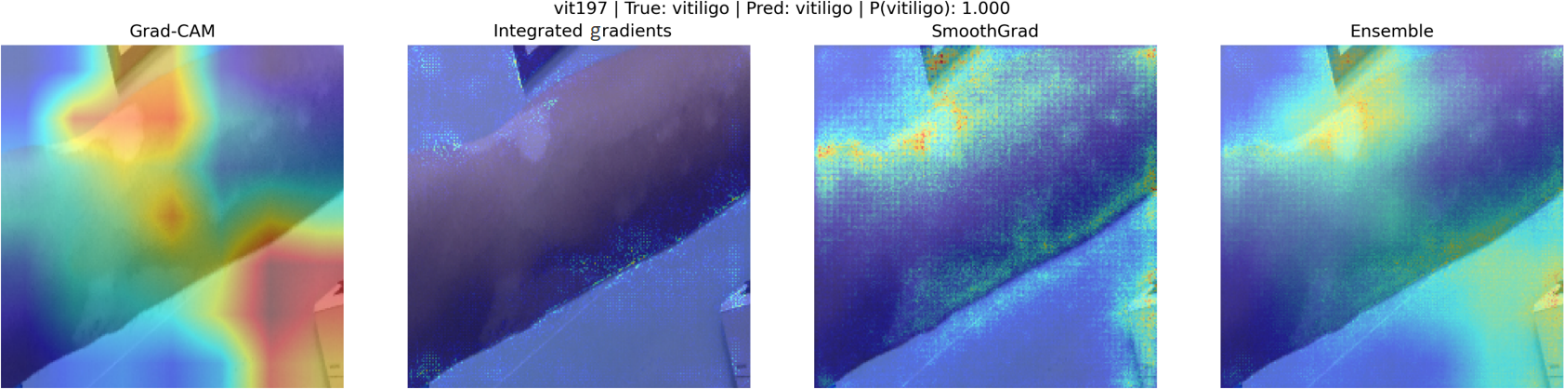
Representative example of scenario 3, where the individual explanation methods emphasized complementary lesion characteristics and the ensemble map integrated them into a unified interpretation. Grad-CAM: gradient-weighted class activation mapping; SmoothGrad: smooth gradients.

**Figure 9.**
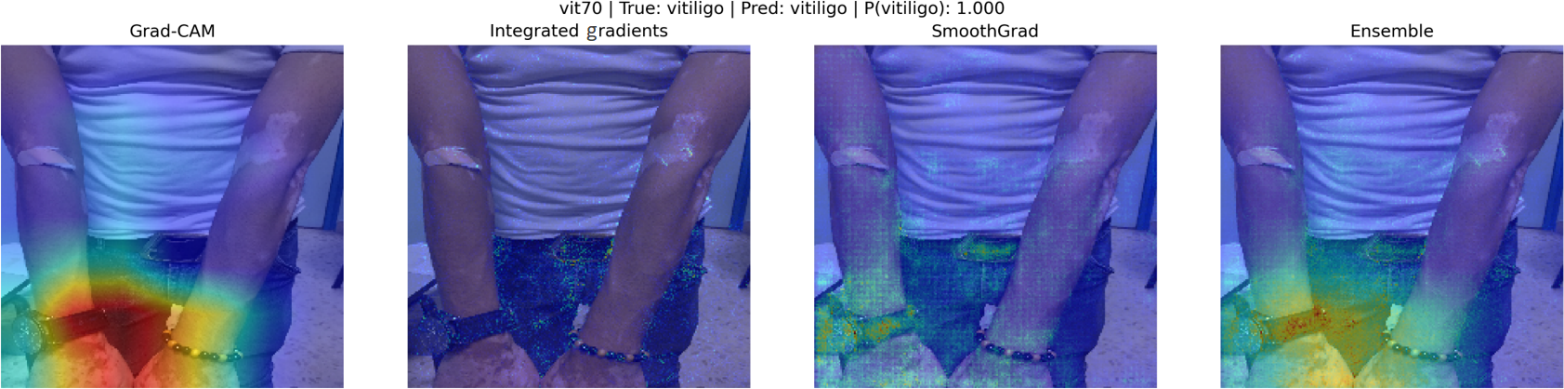
Representative example of scenario 4, where all explanation methods produced noisy or partially inconsistent saliency maps, resulting in a less informative ensemble map. Grad-CAM: gradient-weighted class activation mapping; SmoothGrad: smooth gradients.

Interpretability analysis was performed using Grad-CAM, integrated gradients, SmoothGrad, and the proposed ensemble explanation framework. Four recurring interpretability scenarios were identified based on the degree of agreement and complementarity among the explanation methods. Representative examples are presented in Figures 6-9.

#### Scenario 1: High Agreement Across All Methods

In the most common scenario, all 3 explanation methods generated highly consistent saliency maps, with each method focusing on the same clinically relevant lesion region. The resulting ensemble map (Figure 6) closely matched the individual explanations and reinforced the confidence in the model’s prediction.

This scenario was observed in 36 of 66 (54.55%) validation images, indicating that the 3 methods frequently converged on similar and clinically meaningful attribution patterns.

#### Scenario 2: Failure of a Single Explanation Method

In this scenario (Figure 7), 1 explanation method produced a weak, noisy, or uninformative saliency map, while the other 2 methods successfully localized the lesion. Despite the failure of 1 individual method, the ensemble map remained accurate and clinically meaningful by integrating the informative signals from the remaining methods.

This pattern was observed in 25 of 66 (37.88%) images, demonstrating that the ensemble framework effectively mitigated isolated failures of individual explanation techniques.

#### Scenario 3: Complementary Information Across Methods

In a smaller subset of cases, the 3 explanation methods highlighted different but complementary aspects of the lesion. For example, 1 method emphasized lesion borders, whereas another focused on internal pigmentation changes. By combining these complementary signals, the ensemble map provided a more comprehensive and informative explanation than any single method alone (Figure 8). This scenario was identified in 4 of 66 (6.06%) images.

#### Scenario 4: All Methods Are Noisy or Partially Inconsistent

In 1 challenging case, all 3 explanation methods produced noisy or partially inconsistent saliency maps. The ensemble map remained similarly diffuse, providing limited improvement over the individual methods. Figure 9 illustrates this case.

This scenario occurred in only 1 of 66 (1.52%) images, indicating that the proposed framework generated clinically useful explanations in the vast majority of cases.

### Quantitative Distribution of Interpretability Scenarios

To quantitatively assess the prevalence of these interpretability patterns beyond the representative examples shown in Figures 6-9, Table 4 summarizes the distribution of images across the 4 scenarios within a complete validation fold containing 66 images.

**Table 4.** Distribution of validation images across 4 interpretability scenarios (n=66).

Scenario	Description	Images, n (%)
1	High agreement across all methods	36 (54.55)
2	One method fails, but the ensemble remains robust	25 (37.88)
3	Methods provide complementary information	4 (6.06)
4	All methods are noisy or partially inconsistent	1 (1.52)
Total	—^a^	66 (100)

a Not applicable.

A single validation fold was selected as a representative subset of the dataset. This approach is consistent with established practices in explainable artificial intelligence, where qualitative and case-based interpretation analyses are commonly conducted on representative samples rather than the entire dataset because of computational costs and the inherently qualitative nature of visual assessment [37]. Furthermore, within the 5-fold cross-validation framework, each validation fold constitutes a statistically representative partition of the overall dataset, preserving class balance and lesion variability [16].

As shown in Table 4, the ensemble framework produced clinically meaningful explanations in 65 (98.48%) of 66 images, corresponding to scenarios 1‐3. Scenario 1, representing strong agreement across all methods, was the most frequent outcome. Scenario 2 was also common, demonstrating that the ensemble remained reliable even when 1 individual method failed. Scenario 4 was rare, occurring in only a single image.

## Discussion

### Principal Findings

This study developed and validated an interpretable deep learning framework for differentiating vitiligo from PIH, 2 pigmentary disorders that are often difficult to distinguish because of their overlapping clinical appearance. Using a fine-tuned MobileNetV2 model evaluated with patient-wise 5-fold cross-validation, the proposed framework achieved an overall accuracy of 94.88%, a macroaveraged precision of 94.88%, a recall of 94.84%, and an *F*_1_-score of 94.86%, demonstrating balanced classification performance across both classes, and an AUC of 0.9885.

Although patient-wise partitioning generally provides a more conservative estimate of performance, the revised pipeline also involved the reconstruction of the full preprocessing, metadata organization, fold generation, and model evaluation workflow. Because the original dataset already contained minimal patient overlap (mean 1.14, SD 0.66 images per patient; median 1, IQR 1-1, range 1-9 images per patient), patient-level leakage in the earlier image-wise protocol was likely limited. The observed performance difference, therefore, most likely reflects a combination of revised fold composition, random initialization effects, and improved stability of the rebuilt evaluation pipeline rather than leakage alone.

These results indicate that the model achieved high and well-balanced diagnostic performance for both classes. The aggregated confusion matrix demonstrated that 315 of 332 images were correctly classified, with only 17 misclassifications across all validation folds. This performance is particularly notable given that the evaluation protocol ensured that images from the same patient were never shared between training and validation subsets, thereby eliminating patient-level data leakage and providing a more rigorous estimate of generalization to previously unseen patients.

In addition to strong quantitative performance, the proposed framework incorporated an ensemble explainability strategy that combined Grad-CAM, integrated gradients, and SmoothGrad. The resulting explanation maps consistently highlighted clinically relevant features, including lesion border sharpness, pigment uniformity, and contrast with surrounding skin, indicating that the model based its predictions on diagnostically meaningful visual patterns rather than background artifacts or acquisition-specific cues.

From a clinical perspective, differentiating vitiligo from PIH is inherently challenging, particularly in early-stage lesions or in cases with subtle pigmentary changes. The strong performance achieved under a strict patient-wise validation strategy suggests that the model learned robust lesion-specific features that generalize beyond individual patients and image sources.

### Impact of Fine-Tuning and Domain Adaptation

The initial frozen training phase provided a strong baseline by leveraging generic visual features learned from ImageNet pretraining. However, these features are not optimized to capture the subtle morphological and pigmentary characteristics that distinguish vitiligo from PIH.

Fine-tuning the final 30 layers of MobileNetV2 enabled the network to adapt to dermatology-specific patterns. This adaptation improved both quantitative performance and qualitative localization, as evidenced by the stable convergence behavior observed in the training curves and the enhanced focus on lesion-relevant regions in the saliency maps.

A transient increase in validation loss was observed at the beginning of the fine-tuning phase. This behavior is expected when previously frozen layers are unfrozen and the optimizer state is reinitialized, resulting in a temporary adjustment period before the model converges to a new optimum.

### Cross-Validation Stability and Generalization

The patient-wise 5-fold cross-validation framework demonstrated consistent performance across all folds, supporting the robustness of the proposed approach. Because each validation fold contained images from entirely unseen patients, the resulting metrics provide a conservative and clinically realistic estimate of diagnostic performance.

The use of aggregated predictions across all validation folds further strengthened the analysis by enabling computation of final performance metrics from the complete set of 332 out-of-sample predictions rather than from a single fold. This approach reduced the influence of fold-specific variability and yielded a comprehensive assessment of model behavior.

### Error Analysis

Although the overall performance was highly balanced, a small number of errors remained. Specifically, 9 PIH images were misclassified as vitiligo, and 8 vitiligo images were misclassified as PIH.

These errors likely reflect the substantial clinical overlap between the 2 conditions. Early vitiligo lesions may exhibit partial depigmentation or indistinct borders, whereas PIH lesions may present with sharply demarcated hypopigmentation that closely resembles vitiligo. Such borderline cases can be challenging even for experienced dermatologists.

Despite these difficult cases, the low number of misclassifications and the high AUC of 0.9885 indicate excellent discrimination across a wide range of decision thresholds.

### Interpretability Analysis and Value of the Ensemble Framework

Interpretability analysis demonstrated that the ensemble explanation framework substantially improved the robustness of visual explanations.

A complete validation fold containing 66 images was manually reviewed and categorized into 4 predefined interpretability scenarios. In 36 (54.55%) images, all 3 explanation methods showed strong agreement (scenario 1). In 25 (37.88%) images, 1 method produced a weak or noisy map, but the ensemble remained robust by integrating informative signals from the remaining methods (scenario 2). In 4 (6.06%) images, the methods provided complementary information that enriched the final explanation (scenario 3). Only 1 (1.52%) image exhibited noisy or partially inconsistent maps across all methods (scenario 4).

Overall, the ensemble framework produced clinically meaningful explanations in 65 (98.48%) of 66 images. These findings confirm that combining multiple explanation techniques provides greater stability and reliability than relying on any single method alone.

### Comparison With Prior Work

While explainability techniques such as Grad-CAM have been extensively explored in dermatological AI applications, particularly for skin cancer detection [10,22,32], their application to hypopigmented disorders remains limited. Previous studies have demonstrated the value of visual explanations for building clinical trust. For example, Badhon et al [32] and Shorfuzzaman [34] demonstrated increased trust via saliency maps but rarely benchmarked multiple explanation methods together or addressed conditions such as PIH. These studies typically relied on single explainability methods and did not systematically address their individual limitations or explore multimethod ensemble approaches. In contrast, this study integrates 3 complementary explanation methods into a unified ensemble framework and quantitatively evaluates their robustness through predefined interpretability scenarios.

The use of patient-wise cross-validation and the high diagnostic performance achieved under this stricter protocol further strengthen the clinical relevance of the proposed approach.

### Clinical Implications

The combination of high classification performance and robust interpretability makes the proposed framework particularly attractive for clinical decision support.

The lightweight MobileNetV2 architecture offers computational efficiency suitable for deployment in resource-limited environments, mobile apps, and teledermatology platforms. At the same time, the ensemble explanation maps provide transparent visual evidence that can help dermatologists understand and verify the basis of model predictions.

Rather than replacing clinician judgment, the framework is intended to serve as an assistive tool that supports more consistent and objective differentiation between vitiligo and PIH.

### Limitations

Several limitations should be acknowledged.

First, although the dataset size was sufficient to demonstrate proof of concept, it remains relatively modest for deep learning applications. Larger multicenter datasets would be valuable for further validation.

Second, most images were obtained from a single institution, supplemented by publicly available web images. Although all images were processed using a unified preprocessing pipeline, additional validation on external cohorts would strengthen generalizability.

Third, systematic subgroup analyses based on Fitzpatrick skin type, age, ethnicity, lesion location, and disease duration were not possible because these metadata were not consistently available.

Fourth, the model relied exclusively on image data and did not incorporate clinical information such as lesion history, preceding inflammation, or family history, which may further improve diagnostic performance.

Finally, while the explainability analysis was conducted on a representative validation fold, evaluation across all folds and formal assessment by dermatologists would provide additional evidence regarding explanation quality and clinical usefulness.

### Future Directions

Several directions may extend the present work. First, multicenter studies involving larger and more diverse datasets are needed to validate the model across different imaging conditions, patient populations, and skin phototypes. Collecting structured demographic and clinical variables, such as Fitzpatrick skin type, age, ethnicity, lesion location, and disease duration, would enable subgroup analyses to assess fairness and generalizability.

Second, incorporating structured clinical metadata, including lesion onset, history of preceding inflammation, and family history, may improve diagnostic performance and better reflect real-world dermatological decision-making.

Third, the proposed ensemble explainability framework could be further refined by investigating adaptive weighting strategies and quantitatively validated against expert-annotated lesion boundaries and dermatologist assessments.

Fourth, future studies should compare MobileNetV2 with alternative architectures, including larger convolutional models such as ResNet and EfficientNet [38] and recent vision foundation models [39,40], to evaluate the trade-off between predictive performance and computational efficiency.

Finally, prospective clinical studies are needed to assess the impact of the proposed framework on diagnostic accuracy, clinician confidence, and workflow efficiency, particularly in teledermatology and resource-limited settings.

### Conclusions

This study developed an interpretable deep learning framework for differentiating vitiligo from PIH using a fine-tuned MobileNetV2 model and an ensemble of Grad-CAM, integrated gradients, and SmoothGrad.

Under a rigorous patient-wise 5-fold cross-validation protocol, the model achieved excellent diagnostic performance, with an overall accuracy of 94.88%, an *F*_1_-score of 95.18%, and an AUC of 0.9885. The ensemble explanation framework generated robust and clinically meaningful visual explanations in 98.48% of a representative 66-image validation subset used for interpretability assessment.

These findings demonstrate that combining lightweight deep learning architectures with complementary explainability methods can provide both high diagnostic accuracy and clinical transparency. The proposed framework has strong potential as a decision-support tool for dermatologists, particularly in settings where specialist expertise is limited.

## Supplementary material

10.2196/81942 Multimedia Appendix 1 Training and validation curves for all 5 cross-validation folds during the frozen training and fine-tuning phases, including fold-specific best-validation and stopping epochs.

## Abbreviations

AUC: area under the receiver operating characteristic curve
CAM: class activation mapping
CNN: convolutional neural network
Cycle-GAN: Cycle-Consistent Adversarial Network
DenseNet: Densely Connected Convolutional Networks
Grad-CAM: gradient-weighted class activation mapping
KAUH: King Abdullah University Hospital
PIH: postinflammatory hypopigmentation
ResNet: residual network
ROC: receiver operating characteristic
SHAP: Shapley additive explanations
SmoothGrad: smooth gradients
VGG: Visual Geometry Group
XAI: explainable artificial intelligence
YOLO: You Only Look Once
