# Supplementary material for: Clinically Interpretable Deep Learning for Differentiating Vitiligo and Postinflammatory Hypopigmentation: Diagnostic Accuracy Study
Source: JMIR Med Inform. 2026 Jul 24;14:e81942. doi: 10.2196/81942 (PMC13399407; doi:10.2196/81942)
Supplement: Multimedia Appendix 1 [file medinform-v14-e81942-s001.docx]

### Appendix 1. Training and Validation Curves for All Cross-Validation Folds

This appendix provides the complete set of fold-specific training and validation curves for both the frozen training phase and the fine-tuning phase of the MobileNetV2 model. In each figure, panel (a) presents the training and validation accuracy curves, and panel (b) presents the corresponding training and validation loss curves.


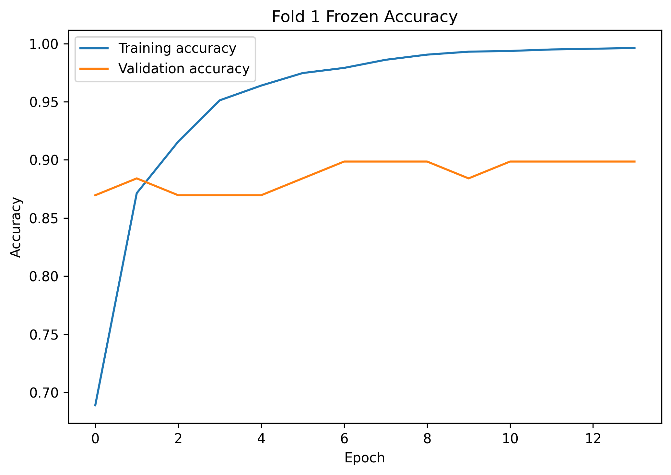

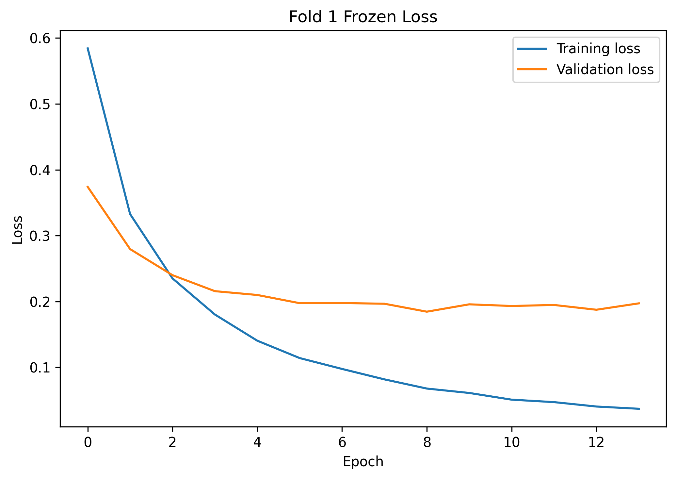


(a) (b)

Figure S1: Training and validation curves for Fold 1 during the frozen training phase. Panel (a) shows training and validation accuracy, and panel (b) shows training and validation loss.


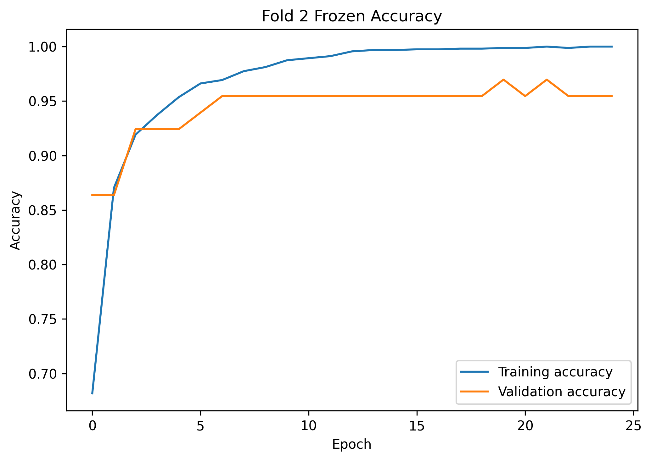

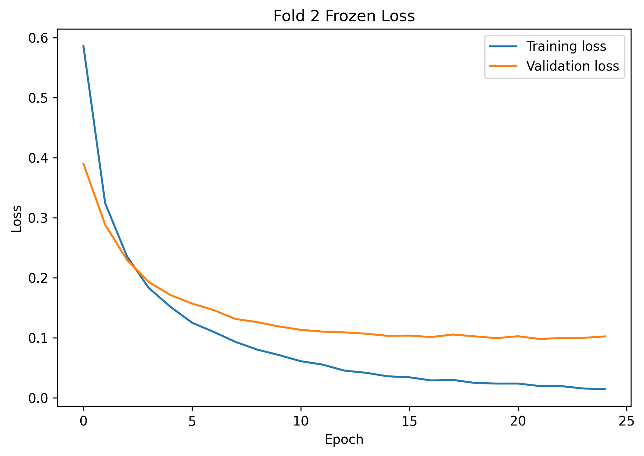


(a) (b)

Figure S2: Training and validation curves for Fold 2 during the frozen training phase. Panel (a) shows training and validation accuracy, and panel (b) shows training and validation loss.


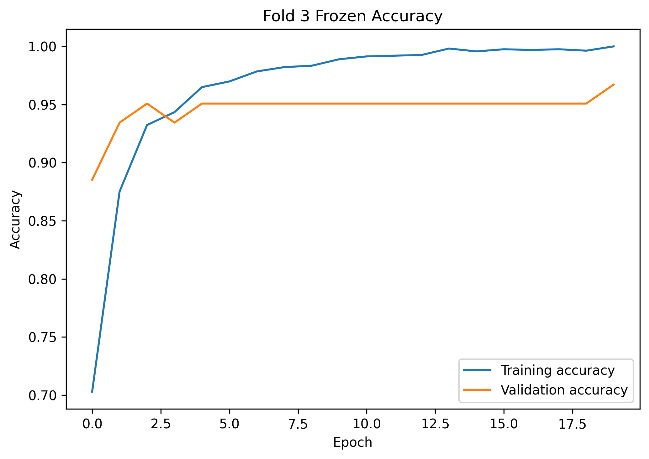

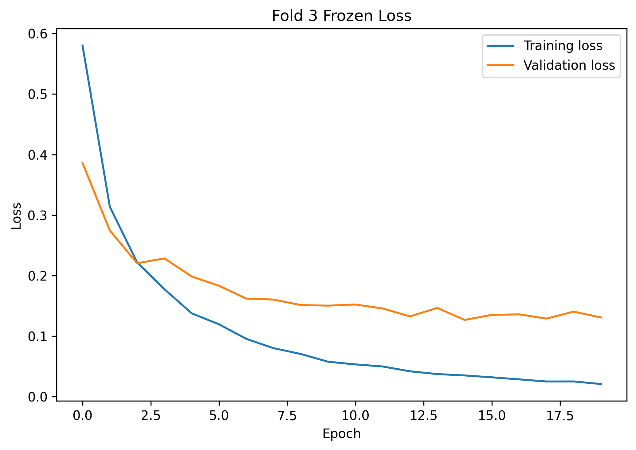


(a) (b)

Figure S3: Training and validation curves for Fold 3 during the frozen training phase. Panel (a) shows training and validation accuracy, and panel (b) shows training and validation loss.


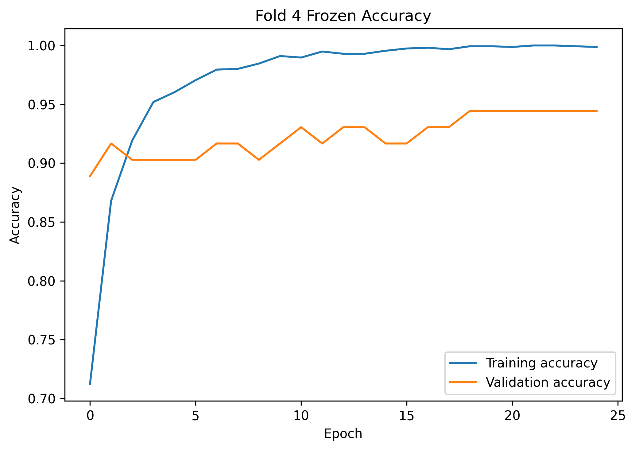

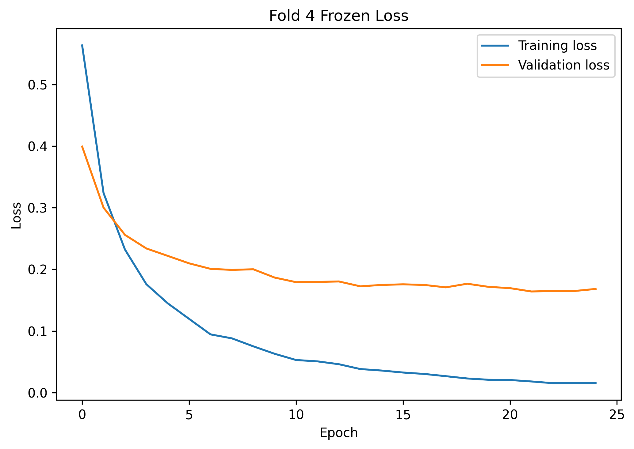


(a) (b)

Figure S4: Training and validation curves for Fold 4 during the frozen training phase. Panel (a) shows training and validation accuracy, and panel (b) shows training and validation loss.


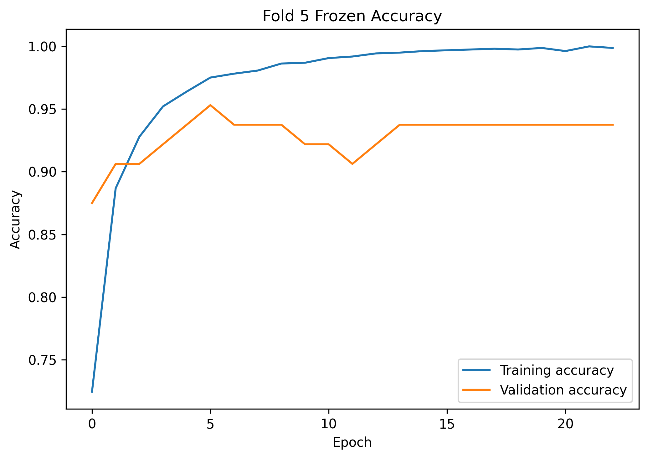

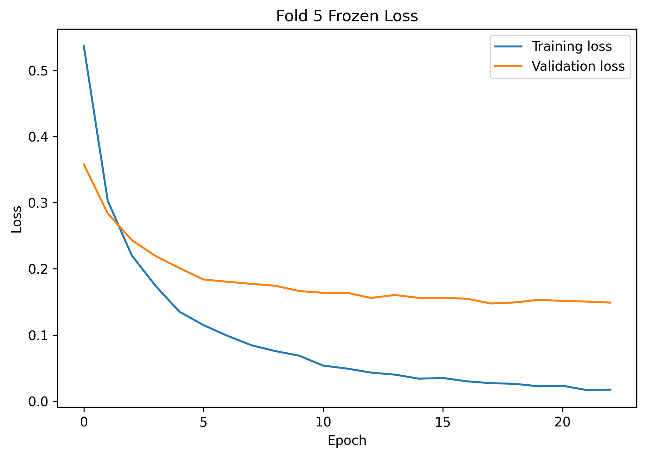


(a) (b)

Figure S5: Training and validation curves for Fold 5 during the frozen training phase. Panel (a) shows training and validation accuracy, and panel (b) shows training and validation loss.


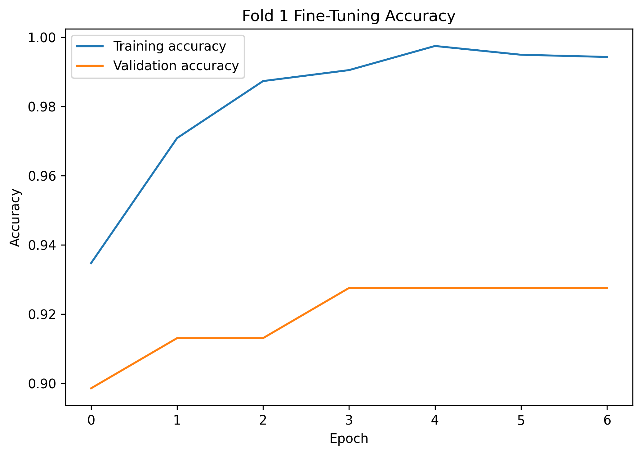

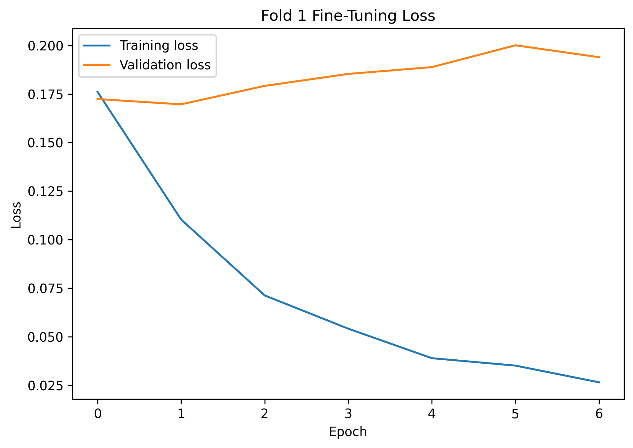


(a) (b)

Figure S6: Training and validation curves for Fold 1 during the fine-tuning phase. Panel (a) shows training and validation accuracy, and panel (b) shows training and validation loss.


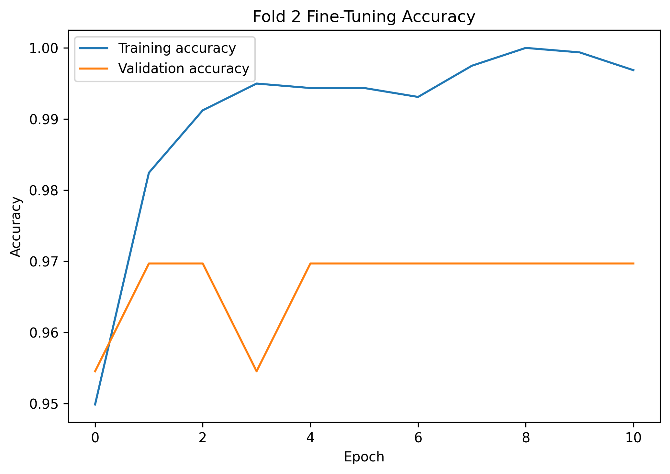

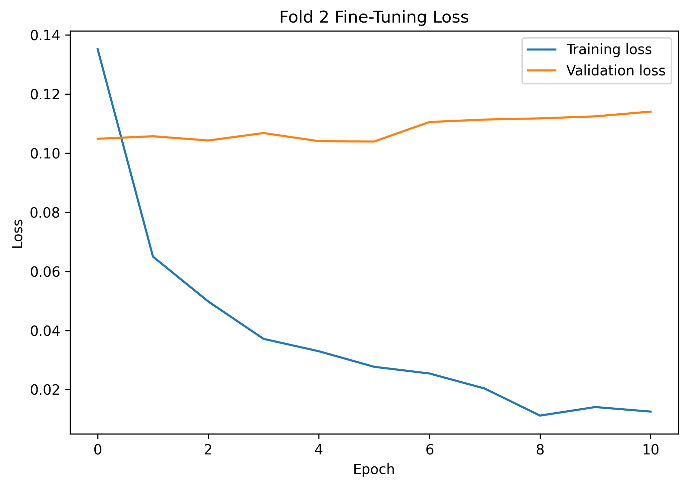


(a) (b)

Figure S7: Training and validation curves for Fold 2 during the fine-tuning phase. Panel (a) shows training and validation accuracy, and panel (b) shows training and validation loss.


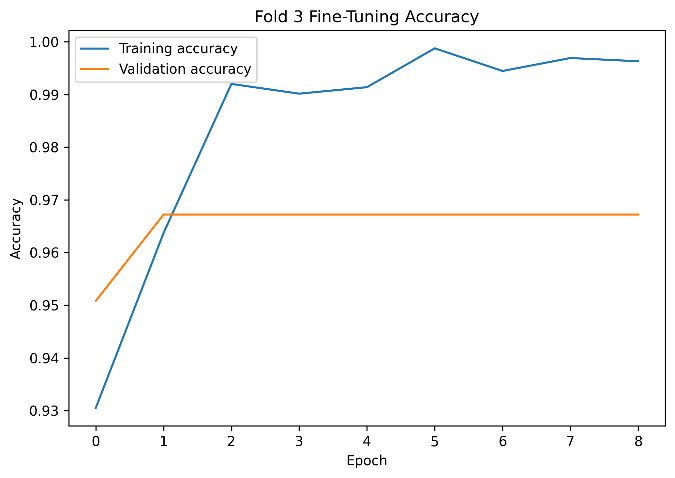

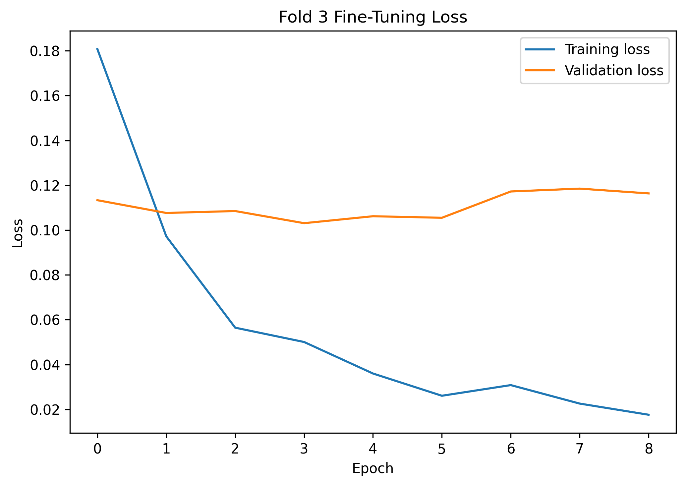


(a) (b)

Figure S8: Training and validation curves for Fold 3 during the fine-tuning phase. Panel (a) shows training and validation accuracy, and panel (b) shows training and validation loss.


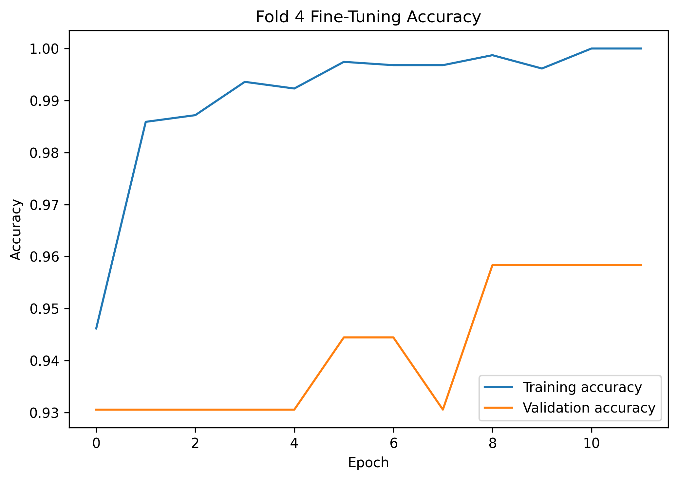

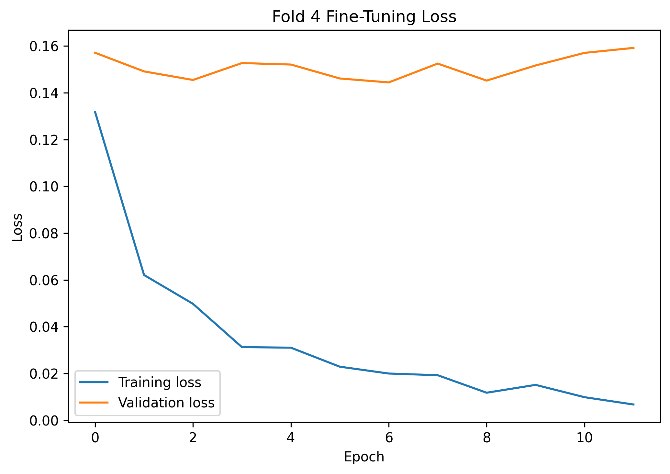


(a) (b)

Figure S9: Training and validation curves for Fold 4 during the fine-tuning phase. Panel (a) shows training and validation accuracy, and panel (b) shows training and validation loss.


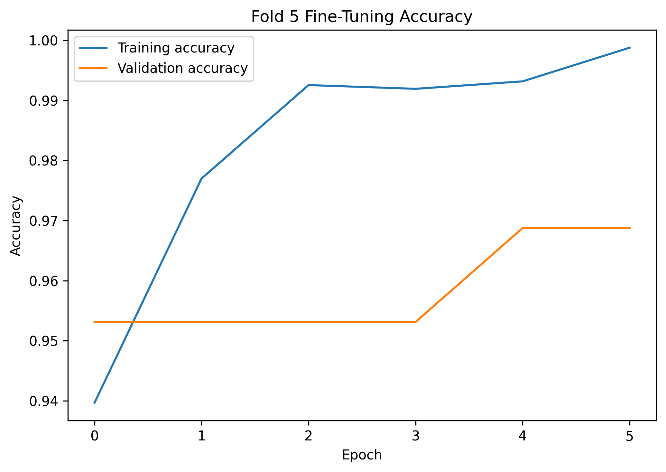

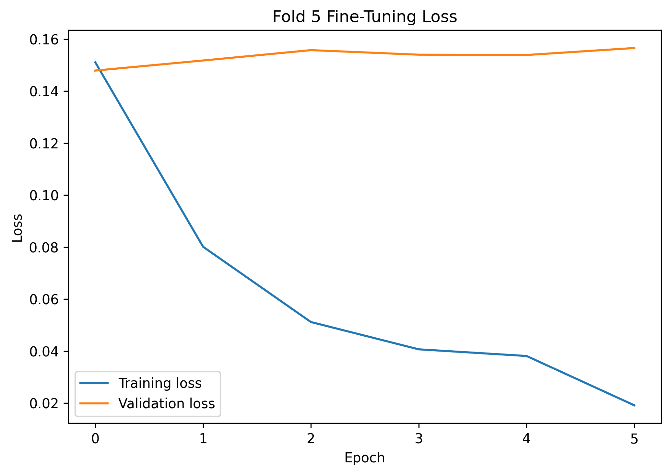


(a) (b)

Figure S10: Training and validation curves for Fold 5 during the fine-tuning phase. Panel (a) shows training and validation accuracy, and panel (b) shows training and validation loss.

**Table S1. Fold-specific best-validation and stopping epochs during frozen training and fine-tuning.**

| **Fold** | **Best Frozen Epoch** | **Frozen Stopping Epoch** | **Best Fine-Tuning Epoch** | **Fine-Tuning Stopping Epoch** |
| --- | --- | --- | --- | --- |
| 1 | 9 | 14 | 2 | 7 |
| 2 | 22 | 25 | 6 | 11 |
| 3 | 15 | 20 | 4 | 9 |
| 4 | 22 | 25 | 7 | 12 |
| 5 | 18 | 23 | 1 | 6 |
